# Liraglutide does not increase heart rate of diabetic patients during acute myocardial infarction

**DOI:** 10.1111/1753-0407.13517

**Published:** 2024-01-03

**Authors:** Qianyi Li, Chunxuan Wu, Shiqun Sun, Lingchao Yang, Yanyan Li, Yixin Niu, Li Zhang, Wei Li, Ying Yu

**Affiliations:** ^1^ Department of Cardiology Xinhua Hospital, School of Medicine, Shanghai Jiao Tong University Shanghai China; ^2^ Department of Endocrinology Xinhua Hospital, School of Medicine, Shanghai Jiao Tong University Shanghai China

**Keywords:** acute myocardial infarction, blood pressure, GLP‐1RA, heart rate, Type 2 diabetes mellitus

## Abstract

**Background:**

Glucagon‐like peptide 1 receptor agonists have been shown to reduce all‐cause and cardiovascular mortality in patients with Type 2 diabetes mellitus (T2DM). The probable increase in heart rate hinders its early usage in acute myocardial infarction patients. In our study, we aimed to find out whether the use of liraglutide in patients with acute myocardial infarction as early as at the time of hospitalization would increase the heart rate.

**Methods:**

This was an observational retrospective study. From December 2020 to August 2021, 200 patients with acute myocardial infarction were included in our study and divided into three groups: T2DM + liraglutide group (*n* = 46), T2DM + non‐liraglutide group (*n* = 42), and non‐T2DM group (*n* = 112). The primary outcomes were the differences in heart rate. Secondary outcomes were differences in systolic and diastolic blood pressure.

**Results:**

There were no significant differences in heart rate among the three groups at admission, the day before the first shot of liraglutide, and before discharge. There was also no significant difference in heart rate between diabetic patients with acute myocardial infarction and those on liraglutide during the hospital stay. And there were no differences of beta‐blocker dosages among the three groups. Liraglutide did not affect the blood pressure during acute myocardial infarction.

**Conclusions:**

Liraglutide did not increase the heart rate in diabetic patients during acute myocardial infarction and did not lead to an increase in the dose of beta‐blockers in the patients. It also had no effect on blood pressure and showed better efficacy in lowering glucose levels without additional hypoglycemic events.

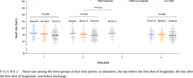

## Graphical abstract

Highlights

- Glucagon‐like peptide 1 receptor agonists have been shown to reduce all‐cause and cardiovascular mortality in patients with Type 2 diabetes mellitus.
- Previous studies have found that liraglutide can increase the heart rate in patients, so the use of liraglutide should be controlled in patients with acute myocardial infarction.
- We found that liraglutide did not increase the heart rate of diabetic patients during acute myocardial infarction and it had no effect on blood pressure.
- Therefore, liraglutide is safe for use in the early stages of acute myocardial infarction.

## 1 INTRODUCTION

Cardiovascular disease, particularly acute myocardial infarction (AMI), is the leading cause of disease‐related mortality in the world.^1^ Compared with nondiabetic patients, diabetic patients have a higher risk of AMI (2‐ to 4‐fold increased) and cardiovascular mortality.^2^, ^3^

As glucose‐lowering agents, glucagon‐like peptide 1 receptor agonists (GLP‐1RA) have also been shown to reduce both all‐cause and cardiovascular mortality in patients with Type 2 diabetes mellitus (T2DM).^4^, ^5^, ^6^ Some experimental studies with AMI animal models and patients have suggested that liraglutide, a GLP‐1RA, could ameliorate coronary no‐reflow during percutaneous coronary intervention and improve left ventricular function.^7^, ^8^, ^9^

However, some studies showed that liraglutide might increase the heart rate (HR) in a dose‐related manner. But this result is controversial in other studies.^10^, ^11^, ^12^ Lovshin et al. found that liraglutide increased the HR of T2DM patients with hypertension or chronic heart failure, but without serious adverse cardiovascular events.^13^, ^14^, ^15^, ^16^, ^17^, ^18^ However, the mechanisms by which GLP‐1RA increase HR are not yet fully understood.

In practice, therefore, the probable increase in HR hinders the early usage of GLP‐1RA in AMI patients. In our study, we aimed to find out whether the use of liraglutide in AMI patients as early as at the time of hospitalization would increase the HR.

## 2 MATERIALS AND METHODS

### 2.1 Study population

This is an observational retrospective study based on clinical data stored in the electronic medical record system of Xinhua Hospital affiliated to Shanghai Jiaotong University School of Medicine. Data of all consecutive patients who were admitted to the cardiac care unit (CCU) of the Department of Cardiology in our hospital for AMI from December 2020 to August 2021 were retrospectively evaluated.

### 2.2 Trial procedures

Excluding patients who died during hospitalization, the other patients were divided into three groups according to diabetes diagnosis and prescription of liraglutide during hospitalization: T2DM + liraglutide group, the AMI patients with T2DM receiving liraglutide; T2DM + non‐liraglutide group, the AMI patients with T2DM not receiving liraglutide; non‐T2DM group, the AMI patients without T2DM. The starting dosage of liraglutide was 0.6 mg per day during the first 3 days and was then increased if necessary. Both the patients in the T2DM + liraglutide group and those in the T2DM + non‐liraglutide group could take other hypoglycemic agents if necessary. All the patients received conventional treatment of AMI.

Data of the patients in all three groups were collected retrospectively. Patients’ anthropometric parameters, including age, sex, body mass index (BMI), and Killip classification, and biochemical indicators such as fasting blood glucose (FBG), 2‐h postprandial blood glucose, glycosylated hemoglobin (HbA1c), and C‐peptide were recorded. Patients' ejection fraction (EF) as the most important echocardiographic parameters; comorbidities such as hypertension, heart failure, atrial fibrillation, cerebral infarction, intracerebral hemorrhage, and chronic kidney disease; and T2DM history, diabetes duration, and medicine use (e.g., antiplatelet drugs, beta‐blockers, angiotensin receptor neprilysin inhibitor [ARNI], statins, and hypoglycemic agents) were also documented. Simultaneously, information about patients' hospitalization was gathered, including the length of stay and the cost of hospitalization. HR, systolic blood pressure (SBP), and diastolic blood pressure (DBP) on the day of admission, the day before and after the first shot of liraglutide (for the T2DM + liraglutide group) or in the middle of hospitalization (for the T2DM + non‐liraglutide group and non‐T2DM group), and before discharge were also collected through the electronic medical record system.

The study protocol was approved by the local ethics committee.

### 2.3 Outcomes

The primary outcomes were the differences in HR among the three groups. Secondary outcomes were differences in SBP and DBP among the three groups.

### 2.4 Statistical analysis

Descriptive data are expressed as mean ± standard deviation or median (range 25%–75%) for continuous variables, and frequencies were used for categorical variables. Differences in continuous variables among groups were compared using one‐way analysis of variance (ANOVA) when the variables showed a normal distribution and the Kruskal–Wallis test when the variables were not normally distributed. Two‐way ANOVA was used for multiple paired continuous variables. The chi‐square test was used for unordered categorical variables and dichotomous variables and the Kruskal–Wallis test for ordered categorical variables. All statistical tests were two tailed, and the statistical significance was set at *p* < 0.05. Statistical analyses were performed using SPSS software version 22.0 (SPSS, Chicago, Illinois, USA).

## 3 RESULTS

**FIGURE 1:**
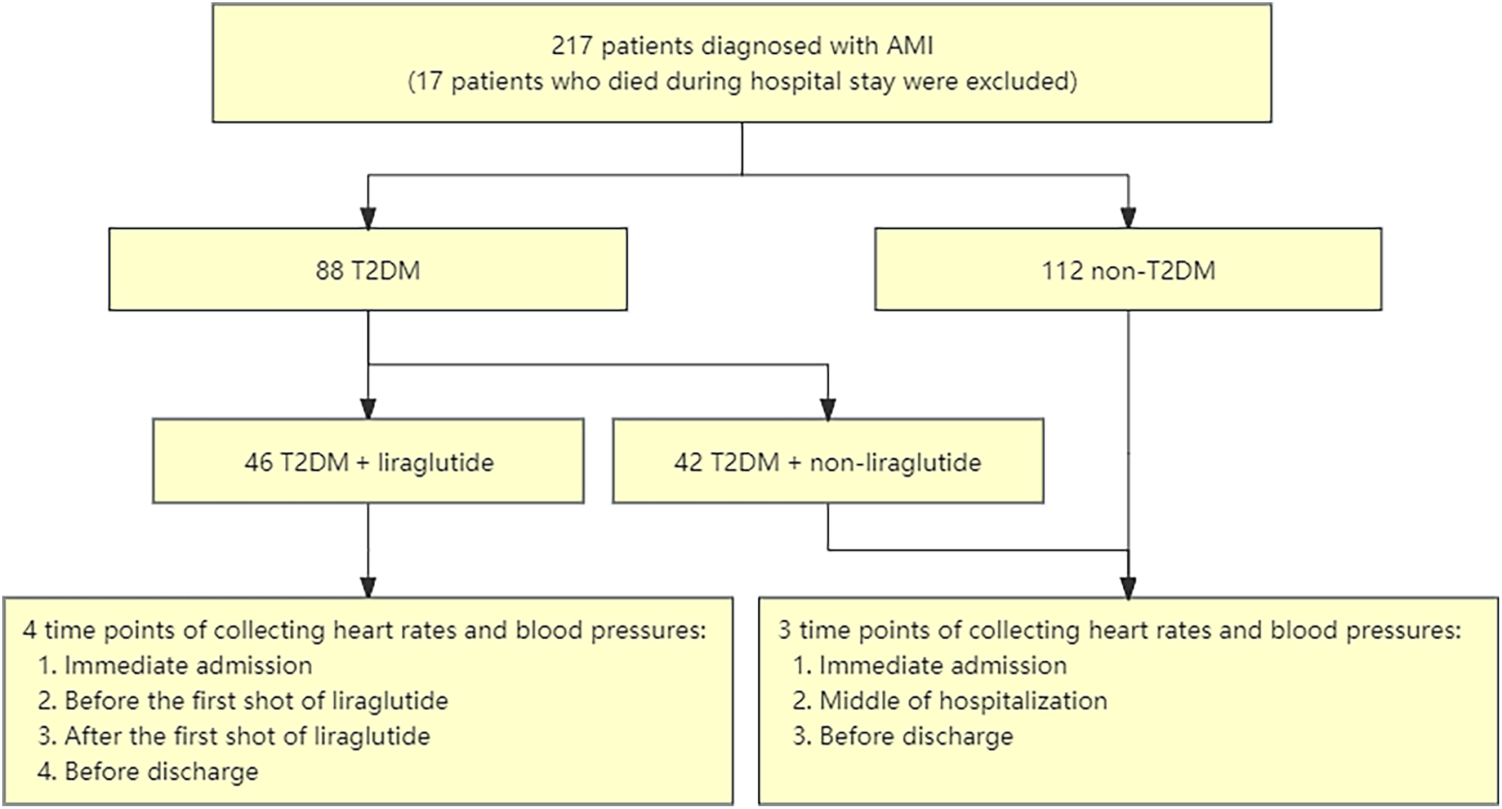
Research flowchart illustrating the inclusion/exclusion process for the study population.

From December 2020 to August 2021, 217 AMI patients were admitted to the CCU. A total of 17 of them died during hospitalization. The other 200 AMI patients (171 males and 29 females) were included in our study. Among them, 46 patients were in the T2DM + liraglutide group, 42 patients in the T2DM + non‐liraglutide group, and 112 patients in the non‐T2DM group. Figure 1 shows an overview of the selection process.

In this study, 200 patients who were admitted to the CCU due to AMI were examined, 17 of whom died during hospitalization and were excluded. Among them, 88 patients had T2DM, and 112 patients did not have T2DM. The diabetic patients were divided into a liraglutide group (46 patients) and a non‐liraglutide group (42 patients) according to whether they used liraglutide during hospitalization. In the diabetic patients, HR and blood pressure were recorded at four time points: admission, the day before the first shot of liraglutide, the day after the first shot of liraglutide, and before discharge. In the nondiabetic patients, HR and blood pressure were taken at three time points: admission, in the middle of hospitalization, and before discharge.

### 3.1 Basic characteristics

The study population was predominantly male (85.5%), with a mean age of 59 years (interquartile range [IQR] 66–73) and a BMI of 23.9 kg/m^2^ (IQR 21.9–26.1). All patients were diagnosed with AMI, of whom 68.5% had an ST‐elevation myocardial infarction and 31.5% a non‐ST‐elevation myocardial infarction. The median EF was 56%. Most patients were treated with antiplatelet drugs (92.5%), statins (95.5%), ARNI (69.5%), and beta‐blockers (67.5%).

In our study, 44% of the patients had T2DM. The median duration of diabetes was 9 years (IQR 1.125–12.75) in the T2DM + liraglutide group and 10 years (IQR 7–20) in the T2DM + non‐liraglutide group (*p* = 0.269). The median FBG at admission was 9.19 mmol/L (IQR 7.56–11.04) in the T2DM + liraglutide group and 7.83 mmol/L (IQR 5.88–9.55) in the T2DM + non‐liraglutide group (*p* = 0.771). The median FBG before discharge was 7.50 mmol/L (IQR 6.50–9.03) in the T2DM + liraglutide group and 7.65 mmol/L (IQR 6.43–9.05) in the T2DM + non‐liraglutide group (*p* = 0.851). The other comorbidities are presented in Table 1.

**TABLE 1:** Participants' basic characteristics by treatment group.

Basic index	All	T2DM + liraglutide	T2DM + non‐liraglutide	Non‐T2DM	*p*
Age (years)	59.0 (66.0–73.0)	56.0 (62.5–67.3)	64.0 (69.5–77.3)	58.3 (66.0–73.8)	0.001
Sex (M/F)	171/29	44/2	34/8	93/19	0.079
BMI (kg/m^2^)	23.9 (21.9–26.1)	24.2 (22.0–26.4)	23.2 (21.9–25.4)	23.9 (21.7–26.3)	0.536
AMI‐related index					
STEMI/NSTEMI	137/63	36/10	27/15	74/38	0.261
Killip (*n*, %)					<0.001
1	167 (83.5)	34 (73.9)	35 (83.3)	98 (87.5)	
2	17 (8.5)	7 (15.2)	4 (9.5)	6 (5.4)	
3	3 (1.5)	2 (4.4)	3 (7.1)	1 (0.9)	
4	13 (6.5)	3 (6.5)	0 (0.0)	7 (6.3)	
EF (%)	56.0 (51.0–60.0)	54.5 (48.0–58.0)	55.5 (47.8–61.0)	56.0 (52.0–60.5)	0.228
Combined diseases (*n*, %)					
HTN	129 (64.5)	36 (78.3)	34 (81.0)	59 (52.7)	<0.001
1	14 (10.9)	5 (13.9)	4 (11.8)	5 (8.5)	
2	48 (37.2)	16 (44.4)	9 (26.5)	23 (39.0)	
3	67 (51.9)	15 (41.7)	21 (61.8)	31 (52.5)	
HF	15 (7.5)	2 (4.4)	4 (9.5)	9 (8.0)	0.621
AF	12 (6.0)	3 (6.5)	4 (9.5)	5 (4.5)	0.493
CI	11 (5.5)	3 (6.5)	3 (7.1)	5 (4.5)	0.763
ICH	1 (0.5)	1 (2.2)	0 (0.0)	0 (0.0)	0.186
CKD	16 (8.0)	9 (19.6)	4 (9.5)	3 (2.7)	0.002*
T2DM‐related index					
FBG at admission^a^ (mmol/L)	6.44 (5.33–8.24)	9.19 (7.56–11.04)	7.83 (5.88–9.55)	5.57 (4.97–6.50)	<0.001^b^
2hPBG (mmol/L)	10.33	15.67	13.03	8.05	<0.001^b^
	(7.73–14.54)	(12.39–17.66)	(10.31–15.47)	(6.79–10.05)	
C‐peptide (nmol/L)	1.20 (0.89–1.79)	1.28 (0.82–1.82)	1.15 (0.82–2.01)	1.19 (0.95–1.71)	0.958
HbA1c (%)	6.20 (5.73–7.48)	8.40 (7.15–9.85)	7.35 (6.60–8.03)	5.80 (5.60–6.15)	<0.001^b^
First diagnosis (*n*, %)		9 (21.4)	10 (21.7)	‐	
Diabetes history (*n*, %)		37 (78.6)	32 (78.3)	‐	
Mean duration of diabetes (years)		9.0 (1.1–12.8)	10.0 (7.0–20.0)	‐	
FBG before discharge^c^ (mmol/L)	‐	7.50 (6.50–9.03)	7.65 (6.43–9.05)	‐	0.851
Hypoglycemia (*n*, %)	0 (0)	0 (0)	0 (0)	0 (0)	
Medications (*n*, %)					
Antiplatelet drugs	185 (92.5)	44 (95.7)	37 (88.1)	104 (92.9)	0.396
Statin	191 (95.5)	44 (95.7)	39 (92.9)	107 (95.5)	0.532
ARNI	139 (69.5)	45 (97.8)	31 (73.8)	70 (62.5)	0.035
Beta‐blocker	135 (67.5)	31 (67.4)	27 (64.3)	77 (68.8)	0.87
11.88 mg/day	65 (48.1)	11 (35.5)	13 (48.1)	41 (53.2)	
23.75 mg/day	51 (37.8)	13 (41.9)	9 (33.3)	29 (37.7)	
35.63 mg/day	1 (0.7)	0 (0.0)	0 (0.0)	1 (1.3)	
47.5 mg/day	15 (11.1)	4 (12.9)	5 (18.5)	6 (7.8)	
95 mg/day	3 (2.2)	3 (9.7)	0 (0.0)	0 (0.0)	
Mean beta‐blocker dosage (mg/day)^d^	11.88 (0.00‐23.75)	11.88 (0.00–23.75)	11.88 (0.00–23.75)	11.88 (0.00–23.73)	0.596
Liraglutide dosage					
0.6 mg/day	‐	33 (71.7)	‐	‐	
1.2 mg/day	‐	9 (19.6)	‐	‐	
1.8 mg/day	‐	4 (8.7)	‐	‐	
Mean liraglutide dosage (mg/day)		0.82			
Hospital costs (¥)	47 672	51 473	47 582	46 988	0.464
	(36123–73 787)	(36819–89 539)	(37542–73 929)	(34709–71 460)	
Hospital stay (days)	11 (9–14.8)	12 (9–16.3)	12 (9–15.3)	11 (9–14)	0.269

*Note*: Data are expressed as median (range 25%–75%) or number (%).

Abbreviations: 2hPBG, 2‐h postprandial blood glucose; AF, atrial fibrillation; AMI, acute myocardial infarction; ARNI, angiotensin receptor neprilysin inhibitor; BMI, body mass index; CI, cerebral infarction; CKD, chronic kidney disease; EF, ejection fraction; FBG, fasting blood glucose; HbA1c, glycosylated hemoglobin; HF, heart failure; HTN, hypertension; ICH, intracerebral hemorrhage; NSTEMI, non‐ST‐elevation myocardial infarction; STEMI, ST‐elevation myocardial infarction; T2DM, Type 2 diabetes mellitus.

^a^ Measured with venous blood at admission.

^b^ The *t* test result was obtained from the comparison between the T2DM + liraglutide group and T2DM + non‐liraglutide group.

^c^ Measured with capillary blood glucose before discharge.

^d^ Standardized beta‐blocker dosage at discharge: normalized to succinate metoprolol if the patients used bisoprolol (10 mg bisoprolol was equal to 190 mg succinate metoprolol in this study).^19^

There were no significant differences of the hospitalization costs and the hospitalization duration among three groups (Table 1).

### 3.2 Heart rate

**FIGURE 2:**
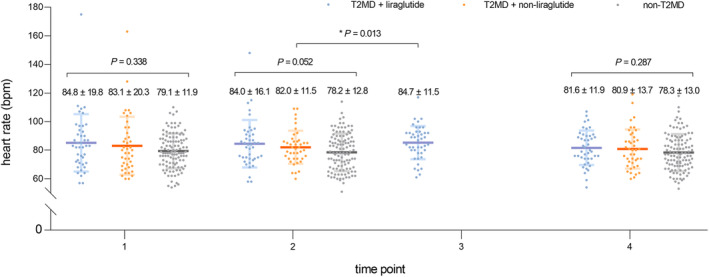
Heart rate among the three groups at four time points: at admission, the day before the first shot of liraglutide, the day after the first shot of liraglutide, and before discharge.

We compared the HR of three groups at four time points: at admission, the day before the first shot of liraglutide, the day after the first shot of liraglutide, and before discharge for the T2DM + liraglutide group and at admission, in the middle of hospitalization (used twice to compare with the HR at the day before and the day after the first shot of liraglutide in the T2DM + liraglutide group), and before discharge for the T2DM + non‐liraglutide and non‐T2DM groups. There were no significant differences of HR among the three groups at admission (84.8 ± 19.8 beats per minute [bpm] in the T2DM + liraglutide group, 83.1 ± 20.3 bpm in the T2DM + non‐liraglutide group, and 79.1 ± 11.9 bpm in the non‐T2DM group; *p* = 0.338), the day before the first shot of liraglutide (84.0 ± 16.1 bpm in the T2DM + liraglutide group, 82.0 ± 11.5 bpm in the T2DM + non‐liraglutide group, and 78.2 ± 12.8 bpm in the non‐T2DM group; *p* = 0.052), and before discharge (81.6 ± 11.9 bpm in the T2DM + liraglutide group, 80.9 ± 13.7 bpm in the T2DM + non‐liraglutide group, and 78.3 ± 13.0 bpm in the non‐T2DM group; *p* = 0.287). There was no significant difference in HR between groups measured at hospital admission, indicating no difference in baseline HR. However, the HR of the T2DM + liraglutide group was slightly higher on the day after the first shot of liraglutide than of those of the other two groups (84.7 ± 11.5 bpm in the T2DM + liraglutide group, 82.0 ± 11.5 bpm in the T2DM + non‐liraglutide group, and 78.2 ± 12.8 bpm in the non‐T2DM group; *p* = 0.013) (Figure 2).

In addition, given that beta‐blockers have a certain HR‐lowering effect, we compared the doses of beta‐blockers in different groups of patients to exclude the effect of beta‐blockers on HR. However, there were no significant differences of beta‐blocker dosages among the three groups (11.88 [IQR 0.00–23.75] mg/day in the T2DM + liraglutide group, 11.88 [IQR 0.00–23.75] mg/day in the T2DM + non‐liraglutide group, and 11.88 [IQR 0.00–23.75] mg/day in the non‐T2DM group; *p* = 0.596; Table 1). There were no significant differences of average hospital stay among the three groups (12 days in the T2DM + liraglutide group, 12 days in the T2DM + non‐liraglutide group, and 11 days in the non‐T2DM group; *p* = 0.269; Table 1). When we compared the HR of each group before and after hospitalization, no significant difference was found in each group. Thus, our results suggest that liraglutide has no effect on HR and does not require an increase in beta‐blockers or longer hospitalization. There are no usual HR control medications other than beta‐blockers during patients' hospitalization.

Patients were divided into three groups according to whether they had T2DM and used liraglutide. The HRs of the patients at four time points of admission and discharge were collected and expressed as mean ± SD.

The *p* values were obtained from one‐way ANOVA on the day after the first shot of liraglutide and before discharge and from a Kruskal–Wallis test at admission and the day before the first shot of liraglutide.

### 3.3 Blood pressure

Interestingly, unlike the HR, the SBP at admission in the two diabetic groups was higher than that in the nondiabetic group (136.1 ± 21.0 mm Hg in the T2DM + liraglutide group, 137.7 ± 20.2 mm Hg in the T2DM + non‐liraglutide group, and 126.0 ± 23.7 mm Hg in the non‐T2DM group; *p* = 0.004; Table 2), which was consistent with the higher hypertension morbidity in the two diabetic groups than that in the nondiabetic group (78.3% in the T2DM + liraglutide group, 81.0% in the T2DM + non‐liraglutide group, and 52.7% in the non‐T2DM group; *p* = 0.003; Table 1). These differences still existed before discharge among the three groups (126.4 ± 14.3 mm Hg in the T2DM + liraglutide group, 127.9 ± 19.8 mm Hg in the T2DM + non‐liraglutide group, and 119.6 ± 19.1 mm Hg in the non‐T2DM group; *p* = 0.004; Table 2). However, there were no significant differences between the SBP values of the two diabetic groups. Our results did not show any difference in DBP among these three groups.

**TABLE 2:** SBP and DBP among three groups.

	T2DM + liraglutide		T2DM + non‐liraglutide		Non‐T2DM		*p*	
Time point^a^	SBP	DBP	SBP	DBP	SBP	DBP	*p* ^SBP^	*p* ^DBP^
1	136.1 ± 21.0	75.4 ± 13.5	137.7 ± 20.2	73.9 ± 10.5	126.0 ± 23.7	73.2 ± 13.8	0.004^b^	0.452
2	124.6 ± 16.3	75.5 ± 11.4	123.5 ± 16.4	70.4 ± 14.0	119.0 ± 18.6	70.0 ± 13.8	0.18	0.057
3	126.6 ± 16.3	74.8 ± 10.0	‐	‐	‐	‐	0.087	0.095
4	126.4 ± 14.3	74.3 ± 9.7	127.9 ± 19.8	69.5 ± 11.5	119.6 ± 19.1	68.8 ± 12.1	0.004^b^	0.038

Abbreviations: DBP, diastolic blood pressure; SBP, systolic blood pressure; T2DM, Type 2 diabetes mellitus.

^a^ Four time points: at admission, the day before the first shot of liraglutide, the day after the first shot of liraglutide, and before discharge.

^b^ *p* values have been obtained from one‐way analysis of variance among the three groups.

Our findings showed that there were no differences in SBP (136.1 ± 21.0 mm Hg at admission, 124.6 ± 16.3 mm Hg on the day before the first shot of liraglutide, 126.6 ± 16.3 mm Hg on the day after the first shot of liraglutide, and 126.4 ± 14.3 mm Hg before discharge; *p* = 0.707) or DBP (75.4 ± 13.5 mm Hg at admission, 75.5 ± 11.4 mm Hg on the day before the first shot of liraglutide, 74.8 ± 10.0 mm Hg on the day after the first shot of liraglutide, and 74.3 ± 9.7 mm Hg before discharge; *p* = 0.530) levels at these four time points in the T2DM + liraglutide group.

## 4 DISCUSSION

### 4.1 Liraglutide does not increase HR in AMI patients

In our study, we did not find any difference in HR among the three groups at admission, before the first shot of liraglutide, and before discharge. Although compared to the other two groups, the patients in the T2DM + liraglutide group showed a slight increase in HR after the first shot of liraglutide (*p* = 0.013). There was no significant difference in HR between before and after hospitalization in the same group, but the HR from admission to discharge in each group had a decreasing trend. Since the time of starting liraglutide was not consistent, the HR of the patients may be related to the length of stay. Taken together, our results suggest that liraglutide does not increase the HR in diabetic patients during AMI.

Rondinelli et al. found that the use of liraglutide in diabetic patients did not increase the patients' HR.^10^, ^20^ However, most of the patients in that study did not have related heart diseases. However, Tougaard et al. found that liraglutide increased the HR about 8–9 bpm in patients with systolic heart failure (average left ventricular ejection fraction [LVEF] 34%–35%).^10^, ^13^, ^17^, ^18^, ^21^, ^22^ We supposed that this difference was influenced by heart function. The average LVEF of patients in our study was 56%, which was better than theirs. These results indicate that liraglutide might cause the HR augment in patients with heart diseases, especially with low LVEF. In these circumstances, we should use liraglutide with caution because an obvious HR increase would burden myocardial oxygen consumption, leading to myocardial ischemia or heart failure. Increased HR has also been found with liraglutide in people with diabetes, mostly in longer‐range studies with a mild increase in HR at 1–3 years of follow‐up.^4^, ^13^, ^23^ In some studies, elevated HR has been found to correlate with the dose of liraglutide used. Our study focused on the short‐term management of patients in the early stages of AMI, and the duration of treatment differed from these studies, resulting in inconsistent changes in HR.

### 4.2 Liraglutide did not increase dosages of beta‐blockers

Beta‐blockers are recommended for AMI patients to prevent and treat ventricular remodeling and sudden death.^24^ Interestingly, in our study, 67.5% of patients received succinate metoprolol (a cardioselective beta‐1‐blocker), and there were no differences of beta‐blocker dosages among the three groups (*p* = 0.596). This suggested in another way that the increase in HR after the first liraglutide shot was slight and transient and could recover to the users' own level spontaneously. There was no need to augment the beta‐blockers dosage to prevent this kind of HR increase. Furthermore, Vukotic et al. found that the noncardioselective beta‐blocker propranolol failed to prevent liraglutide‐related HR elevation in patients with cirrhosis and diabetes.^25^

### 4.3 Liraglutide did not affect blood pressure

In our study, SBP in the two diabetic groups was higher than that in the nondiabetic group (*p* = 0.004), which is consistent with the higher hypertension morbidity in the two diabetic groups (*p* = 0.003). However, there were no differences between the two diabetic groups at any time point during the whole hospitalization. This suggests that liraglutide did not affect blood pressure during AMI.

### 4.4 Low‐dosage liraglutide was effective and safe in AMI patients

In view of the short duration, 71.7% of the patients in our study received only 0.6 mg liraglutide per day. Nevertheless, there were no significant differences in fasting glucose levels between the two diabetic groups before discharge. Furthermore, low‐dosage liraglutide had already shown better glucose lowering as far as 1.51 mmol/L in absolute value. There were no hypoglycemia events during liraglutide usage. This suggests that liraglutide can be used safely during AMI and might offer patients a better way to control glucose levels. At the same time, the use of liraglutide did not prolong hospital stay or increase expenses.^20^

### 4.5 Limitations

This study was retrospective. Patients’ choice of liraglutide treatment may have been influenced by personal bias of the doctors in charge. Our observation was limited to the patients' hospitalization, and the dosages of liraglutide had not been titrated to the maximum in most patients before discharge, which could neglect the dose‐dependent effect of liraglutide treatment on HR.

## 5 CONCLUSION

Liraglutide not only did not increase the HR in AMI patients but also did not affect blood pressure. Liraglutide had a better efficiency on glucose lowering without additional hypoglycemic events.

In conclusion, liraglutide can be used safely in most diabetic patients during AMI, except in patients with low LVEF. GLP‐1RA can reduce major adverse cardiovascular events in diabetic patients. The use of liraglutide in AMI patients as early as at the time of hospitalization or before discharge might bring greater benefits to these patients at very high risk of secondary arteriosclerotic cardiovascular disease. More and further explorations are needed.

## FUNDING INFORMATION

This work was supported by the National Natural Science Foundation of China (grant number 81770321).

## CONFLICT OF INTEREST STATEMENT

The authors declare no conflicts of interests.
